# Low Dietary Betaine Intake Is Associated with Increased Blood Cholesterol in Mexican Subjects

**DOI:** 10.3390/healthcare12080819

**Published:** 2024-04-11

**Authors:** Omar Ramos-Lopez, Alma Santuario-Loera

**Affiliations:** Faculty of Medicine and Psychology, Autonomous University of Baja California, Tijuana 22390, Baja California, Mexico; santuario.alma@uabc.edu.mx

**Keywords:** dietary betaine, cholesterol, cardiovascular risk, Mexican population

## Abstract

Background: Betaine, an osmolyte derivative of the metabolite choline and the amino acid glycine, acts as a methyl donor in the conversion of homocysteine to methionine and is involved in the maintenance of adequate lipid metabolism. There is growing evidence for the role of betaine in the development of various lipid-related diseases, including dyslipidemia and cardiovascular risk. This study aimed to analyze associations between betaine intake and blood lipid profiles in Mexican subjects. Methods: A total of 212 adults were randomly recruited in the city of Tijuana, Baja California, Mexico. Betaine intake was estimated using Nutritionist Pro software. Body composition and metabolic measurements were obtained by conventional methods. In the total sample, the average intake of betaine was 14.32 mg/d. Individuals were categorized into three groups according to tertiles of betaine consumption: tertile/group 1 (<4.16 mg/d), tertile/group 2 (4.16–12.02 mg/d), and tertile/group 3 (>12.02 mg/d). Results: Compared to group 3, subjects within group 1 had higher serum levels of total cholesterol (*p* = 0.001), LDL-c (*p* = 0.026), and non-HDL-c (*p* = 0.021). In addition, significant negative Pearson correlations were found between betaine intake and the serum levels of total cholesterol (r = −0.432, 95% CI, −0.684, −0.185, *p* = 0.001), LDL-c (r = −0.370, 95% CI, −0.606, −0.134, *p* = 0.002), and non-HDL-c (r = −0.351, 95%CI, −0.604, −0.098, *p* = 0.007). Conclusions: Our results show that a low intake of betaine is associated with elevated blood cholesterol levels in Mexican subjects. On this basis, betaine consumption could be used as an additional dietary measure for cardiovascular care. However, additional studies are required to confirm our results in other Mexican regions as well as in other populations worldwide.

## 1. Introduction

Betaine, a methyl derivative of the amino acid glycine, is an important nutrient for human health [1]. Betaine is naturally present in foods such as cereal grains, spinach, sugar beets, shellfish, and shrimp [2]. In addition, betaine is endogenously synthesized by the metabolism of choline in the liver and kidney [3]. Betaine is mainly absorbed in the small intestine, and widely distributed via amino acid transport systems, where it is later utilized in metabolic pathways and not excreted [4]. Betaine acts as an osmotic regulator in most tissues, helping to maintain intracellular osmotic pressure and protecting cells, proteins, and enzymes from environmental stressors [5]. Due to its particular chemical structure, betaine also functions as a methyl donor in the conversion of homocysteine (a toxic compound) to methionine, which is catalyzed by the enzyme betaine–homocysteine methyltransferase as part of the one-carbon metabolism cycle [6].

Betaine is involved in the regulation of various metabolic processes, including lipid homeostasis [7]. In this sense, betaine enhances hepatic cholesterol synthesis, conversion of bile acid from cholesterol, and bile acid efflux into the intestine [8]. In addition, betaine reduces the accumulation of triglycerides in the liver by inhibiting lipogenesis and promoting the oxidation of fatty acids and intramuscular fat deposition [9,10]. Consequently, betaine deficiency may contribute to the development of lipid metabolism disorders such as dyslipidemia and cardiovascular disease [11]. However, studies investigating the effects of dietary betaine intake on cardiovascular risk factors are inconsistent. For instance, higher betaine intake was associated with a non-linear increase in the risk of coronary heart disease in African-American participants [12]. A higher prevalence of hypercholesterolemia was also found in healthy individuals with higher betaine consumption [13]. Conversely, regular betaine intake was not associated with cardiovascular risk in postmenopausal women [14]. In Japanese men and women, there was no clear evidence of a link between betaine intake and the risk of death attributed to cardiovascular disease [15]. Meanwhile, it has been postulated that long-term betaine intake could be a protective factor for cardiovascular mortality [16]. Similarly, lower serum low-density lipoprotein cholesterol (LDL-c) concentrations were associated with higher betaine intake in obese individuals [17]. Hence, further studies are needed to clarify the role of betaine consumption in these lipid changes.

Of note, it has been reported that the Mexican population is highly susceptible to the development of dyslipidemia [18]. The most common dyslipidemias in Mexico include hypoalphalipoproteinemia, followed by hypertriglyceridemia and hypercholesterolemia [19]. Dyslipidemia is a major risk factor for coronary heart disease and stroke [20]. In Mexico, an increase in the prevalence and incidence of cardiovascular mortality and its main risk factors, including overweight/obesity, type 2 diabetes, hypertension, and dyslipidemia, has been documented [21]. This fact could be related to the demographic changes that have transcended in our country in recent decades, but also to the adoption of an unhealthy lifestyle characterized by a high consumption of ultra-processed foods rich in refined carbohydrates and saturated fats [22]. Moreover, Mexicans experience important deficiencies in micronutrients, including vitamins and minerals with lipid-lowering antioxidant, and anti-inflammatory properties [23]. Thus, it is of great relevance to promote the consumption of balanced diets to reduce or prevent the development of cardiometabolic risk factors and maintain a better quality of life in the population. Regional diets have been recommended as a means of reducing cardiovascular risk, for example, the Mediterranean diet [24]. In contrast, the traditional Mexican diet, although understudied, has not offered significant cardioprotective benefits [25]. Additional research about the role of particular micronutrients (i.e., betaine) on cardiometabolic risk factors may help to decipher the effects of these compounds in the Mexican diet on cardiovascular health. 

This study aimed to analyze associations between betaine intake and blood lipid profile in Mexican subjects.

## 2. Materials and Methods

### 2.1. Participants

In a cross-sectional design, a total of 212 residents of the city of Tijuana, Baja California, Mexico, were randomly recruited. The sample included apparently healthy adults (aged 18–65 years), of both sexes, who had received a call to participate in this study via social media. Exclusion criteria comprised pregnant or breastfeeding women, smokers, alcohol users, those with a previous diagnosis of diabetes, cardiovascular disease, or thyroid disorder, and those taking lipid-lowering medication or consuming a special diet (nutrient or calorie-restricted) for three months prior to enrollment into the study. This study was approved by the Ethics Committee of the Autonomous University of Baja California (code: D235, approved on 22 October 2019) and complies with the Declaration of Helsinki regarding research involving human subjects. The participants voluntarily signed an informed consent form.

### 2.2. Anthropometrics and Blood Pressure Measurements

Body weight, total body fat, and body mass index (BMI) were automatically determined in a body composition analyzer (Tanita SC-331S, Tanita Corporation, Tokyo, Japan). Waist, hip, and neck circumferences were collected by trained nutritionists following standardized procedures. Briefly, the waist was measured 4 cm above the umbilicus; the hip was measured at the widest part of the hips; whereas the neck was measured between the mid-cervical spine and mid-anterior neck using non-stretchable plastic tape with the participants standing upright. Systolic blood pressure (SBP) and diastolic blood pressure (DBP) were measured in triplicate using an automated sphygmomanometer. 

### 2.3. Dietary Intake and Physical Exercise

Dietary betaine intake was assessed using three 24 h recalls (including two weekdays and one weekend day) to collect information about the habitual diet (type of foods consumed, amounts, and cooking methods) of each volunteer. This dietary instrument has been consistently used in previous and comparable studies exploring relationships between diet and metabolic phenotypes [26,27,28,29]. All foods consumed were registered, including betaine sources such as cereal grains, spinach, sugar beets, shellfish, and shrimp. Food scales and models were shown to participants to increase the accuracy of reported portion sizes. Nutritional supplements were also considered as potential betaine sources. Dietary records were further computed using the Nutritionist Pro software (Axxya Systems, Stafford, TX, USA, https://nutritionistpro.com/ (accessed on 10 January 2024)) in order to obtain the quantity of betaine intake. Mexican foods or culinary preparations that were not listed in the internal Nutritionist Pro database were added using appropriate nutritional equivalences. Subsequently, subjects were categorized in tertiles of betaine consumption in order to clarify the spread of the data, and named alternatively as groups for practical issues: tertile/group 1 (<4.16 mg/d), tertile/group 2 (4.16–12.02 mg/d), and tertile/group 3 (>12.02 mg/d).

Additionally, for exercise assessment, participants were asked if they regularly practiced some type of systematic physical exercise or sport (yes/no) at least 150–300 min a week, as described elsewhere [30].

### 2.4. Biochemical Tests

Blood samples were taken by venipuncture and then centrifuged for laboratory processing. Fasting glucose, total cholesterol, triglycerides, and high-density lipoprotein cholesterol (HDL-c) were determined in a fully automated analyzer (Mindray BS-200, Shenzhen, China) using commercial kits. For quality control, combined human and commercial control serums were used to lower the biochemical measures’ error rate. LDL-c was calculated as total cholesterol—HDL-c—triglycerides/5. Non-HDL-c was estimated as total cholesterol—HDL-c. 

### 2.5. Statistical Analyses

The sample size was based on a previous study exploring associations between dietary intake and body adiposity and metabolic markers [26]. A web tool was used to increase veracity (https://www.surveymonkey.com/mp/sample-size-calculator/ (accessed on 10 January 2024)), assuming a reliability of 95% (α = 0.05) and a statistical power of 80% (β = 0.20). This formula resulted in 205 subjects. A Cohen’s d value was also calculated using an online source (https://lbecker.uccs.edu/ (accessed on 10 January 2024)) taking into account the difference in the levels of total cholesterol values between groups, tertile 1 (202 ± 37) vs. tertile 3 (180 ± 31), resulting in 0.64 (medium effect size). Continuous and categorical variables were represented as means ± standard deviations and as number of cases, respectively. Comparisons of anthropometric and biochemical variables by betaine groups were estimated by ANOVA and applying post hoc (Bonferroni) tests where appropriate to define intergroup differences, whereas sex was screened by a chi-square test. Pearson correlations were run to analyze the associations between betaine consumption and anthropometric and biochemical parameters, where age, sex, BMI, and calorie intake were used as covariates. Correlation analysis was used to determine whether and how strongly two continuous variables are related, assuming that all the observations are independent of each other and that no causality is inferred [31]. Correlation coefficients range from −1 to +1, where 0 indicates no linear or monotonic association, and the relationship becomes stronger as the coefficient approaches an absolute value of 1 [32]. Statistical tests were fitted in the statistical package IBM SPSS 20 (IBM Inc., Armonk, NY, USA). Correlation plots were created using the GraphPad Prism^®^ software, version 6.0C (La Jolla, CA, USA). A *p*-value lower than 0.05 was established as statistically significant.

## 3. Results

In the total sample, the average intake of betaine was 14.32 mg/d. Table 1 shows the characteristics of participants depending on groups of betaine consumption. No significant statistical differences were observed in age, sex, anthropometric, or clinical variables between groups (Table 1). 

The analysis of the intakes of macronutrients by betaine groups revealed no significant differences concerning the average intake of total calories, proteins, fats, carbohydrates, and fiber (Table 2).

The biochemical profile of the population categorized by betaine intake evidenced more serum levels of total cholesterol, non-HDL-c, and LDL-c in volunteers within groups 1 and 2 compared to those in group 3, whereas no significant differences were found for serum glucose and triglycerides (Table 3).

**Figure 1:**
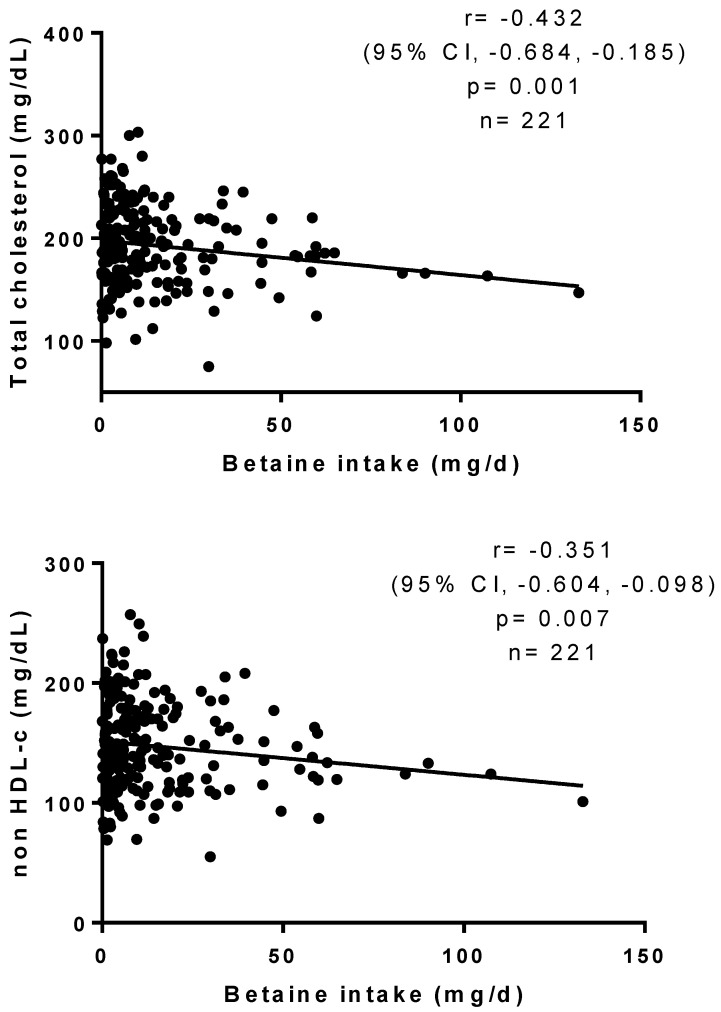

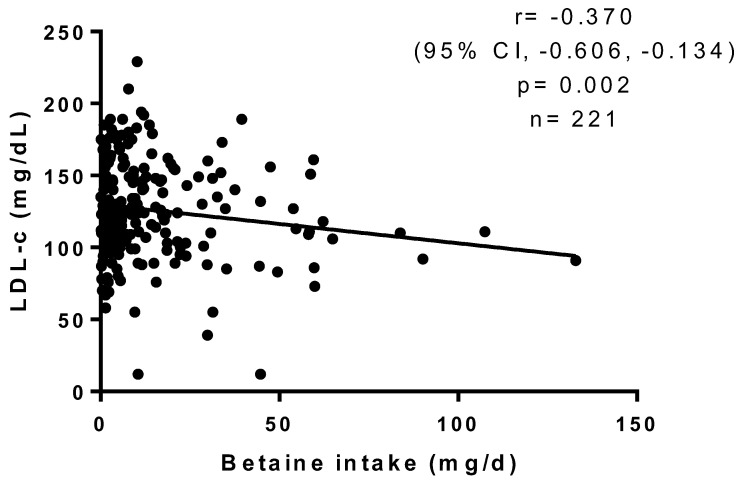
Pearson correlations between betaine consumption and cholesterol parameters. *p*-values are adjusted by age, sex, BMI, and calorie intake.

Adjusted Pearson correlations between betaine consumption and blood cholesterol parameters were plotted (Figure 1). Betaine intake negatively correlated with total cholesterol (r = −0.432, 95% CI, −0.684, −0.185, *p* = 0.001), non-HDL-c (r = −0.351, 95% CI, −0.604, −0.098, *p* = 0.007), and LDL-c (r = −0.370, 95% CI, −0.606, −0.134, *p* = 0.002), with age, sex, BMI, and calorie intake used as covariates.

## 4. Discussion

In this study, an association was evidenced between low betaine consumption and high blood cholesterol levels. This finding suggests that betaine deficiency could contribute to cardiovascular risk by disrupting cholesterol homeostasis. Consistently, higher betaine/choline intake was associated with lower blood pressure and LDL-c concentrations in obese individuals [17]. According to epidemiological studies, long-term choline and betaine intakes may decrease cardiovascular mortality by lowering inflammation and homocysteine levels [16]. However, a higher proportion of hypercholesterolemia was linked to a higher intake of betaine in healthy adults [13]. Moreover, plasma betaine negatively correlated with non-HDL-c and triglycerides in individuals suffering acute coronary syndrome episodes [33]. Likewise, decreased plasma betaine concentrations were linked to an unfavorable cardiovascular risk factor profile (based on blood lipids) in middle-aged and older men and women [34].

The nutritional management of dyslipidemia (including hypercholesterolemia) is mainly based on reducing saturated fat and increasing fiber, unsaturated fatty acids, and plant protein in the diet [35], whereas the role of micronutrients remains less explored. Current evidence suggests that encouraging betaine consumption would favor a beneficial lipid profile and promote cardiovascular health. However, there have been conflicting findings in certain clinical trials concerning the effects of betaine supplementation on blood lipids. In this sense, obese people who followed a hypocaloric diet and supplemented it with betaine (6 g/d for 12 weeks) had higher serum total and LDL-c concentrations [36]. Similarly, betaine therapy (3300 mg twice/d for 10 days, then 4950 mg twice/d for 12 weeks) increased plasma total cholesterol levels in subjects with obesity and prediabetes [37]. Additionally, betaine supplementation (6 g/d for 6 wk) increased blood triglyceride and LDL-c concentrations in healthy individuals [38]. According to a systematic review and meta-analysis, betaine administration (at least 4 g/d for at least 6 weeks) may moderately increase plasma total cholesterol [39]. In contrast, a 3-week betaine supplementation (2.5–5.0 g/d) had no impact on the lipid profile of males who were physically active [40]. Although it has been effective in lowering plasma homocysteine levels, a recognized cardiovascular risk factor, betaine may exert a counterbalancing effect on blood lipids [41]. To address this side effect, the benefit of prescribing low dosages of betaine (<4 g/d) has been suggested as a way to lower homocysteine levels without raising blood lipid levels, as was found with higher doses [42]. 

Animal studies have also reported conflicting findings on the effects of betaine on blood lipids. For instance, 1% betaine supplementation increased blood cholesterol and triglyceride levels in male rats fed a high-fat diet (HFD) for three weeks [43]. In addition, betaine-treated hens (0.5% for 30 days) had greater plasma concentrations of total cholesterol and HDL-c [44]. Serum levels of cholesterol and LDL-c were also higher in laying hens fed a betaine-supplemented (0.5%) diet [45]. Moreover, ApoE-deficient animals given betaine (1–4%) for 14 weeks exhibited greater blood levels of total cholesterol and LDL-c [46]. On the contrary, mice fed HFD and supplemented with 1% betaine showed notable reductions in triglycerides, cholesterol, and LDL-c, along with a minor rise in plasma HDL-c [47]. Furthermore, total cholesterol and triglycerides were reduced after betaine intervention (600–2400 mg/kg) in geese [48]. Based on existing literature, more research is still required to estimate a more accurate dosage and timing of betaine supplementation for a more objective cardiovascular prophylaxis.

The putative mechanisms underlying the effects of betaine on lipid metabolism included changes in gut microbiota (i.e., reductions in *Intestinimonas*, *Acetatifactor*, and *Desulfovibrio*, and increases in *Lachnoclostridium* and *Romboutsia*), with intergenerational metabolic impacts [49]. In addition, maternal betaine supplementation has been shown to reduce hepatic triglyceride content in offspring mice by decreasing the relative abundances of *Proteobateria*, *Desulfovibrio*, and *Ruminococcus*, but increasing *Bacteroides* and *Parabacteroides* [50]. In addition, betaine alleviated diet-induced disruption of hepatic lipid metabolism in mice, which was related to the modification of methylation on lipogenic genes [51]. Moreover, maternal dietary betaine supplementation modified the hepatic expression of cholesterol metabolic genes via alterations in DNA and histone methylation and in the expression of related microRNAs [52]. 

The traditional Mexican diet could be specifically promoted in Mexico due to its nutritional wealth and its variety of healthy foods including grains and tubers, legumes, and vegetables [53]. Indeed, it has been reported that the Mexican diet may decrease cholesterol concentrations in Mexican adults [54]. However, the potential of this dietary pattern to reduce non-communicable diseases including cardiovascular risk remains understudied. Because the traditional Mexican diet includes good sources of betaine, the findings in this research could contribute to delving deeper into the effect of this diet on cardiovascular health, with special emphasis on the role of betaine on blood cholesterol homeostasis.

The strengths of this investigation include the use of appropriate software for the estimation of the betaine of each food consumed. On the other hand, some drawbacks of this study include the biases inherent to cross-sectional studies and correlation analyses (no causality inferred) and the fact that serum betaine concentrations were not determined. In addition, both type I and type II errors cannot be completely discarded despite statistical settings. Furthermore, random recruitment of people at different stages of cardiovascular risk (young and old, male and female with variable BMIs) could represent a limitation of this research, but comparison analyses by betaine groups revealed no differences regarding age, gender, and BMI, as well as correlation analyses were adjusted by these same covariates. In addition, estimations of choline intake and its role in lipid metabolism need to be analyzed further. 

## 5. Conclusions

The results of this study suggest that a low intake of betaine is associated with increased blood cholesterol in Mexican subjects. Based on this, betaine consumption might be used as an additional dietary measure for cardiovascular care. However, additional studies are required to confirm our results in other Mexican regions as well as in other populations worldwide. 

## Figures and Tables

**Table 1:** Characteristics of the total population stratified by groups of betaine consumption.

Variable	Group 1 (n = 70)	Group 2 (n = 71)	Group 3 (n = 71)	*p*
Age (years)	35.7 ± 12.5	39.2 ± 12.8	37.9 ± 12.1	0.247
Sex (F/M)	44/26	45/26	41/30	0.749
BMI (kg/m^2^)	27.9 ± 5.4	29.2 ± 6.1	27.9 ± 4.8	0.273
Body fat (%)	35.8 ± 9.0	36.1 ± 9.0	32.6 ± 9.4	0.284
Waist (cm)	89.1 ± 14.6	92.4 ± 15.5	89.3 ± 13.4	0.319
Hip (cm)	105 ± 10	107 ± 11	103 ± 9	0.120
Neck (cm)	37.1 ± 8.7	36.4 ± 4.4	36.4 ± 4.2	0.796
SBP (mmHg)	120 ± 17	118 ± 20	124 ± 16	0.163
DBP (mmHg)	78.3 ± 9.5	78.5 ± 10.6	79.1 ± 10.3	0.894
Habitual exercise (Yes, %)	58.6	50.7	54.9	0.643

Values are presented as means ± standard deviations. Sex is represented as number of cases. F: females; M: males; BMI: body mass index; SBP: systolic blood pressure; DBP: diastolic blood pressure.

**Table 2:** Nutritional characteristics of the total population stratified by groups of betaine consumption.

Variable	Group 1 (n = 70)	Group 2 (n = 71)	Group 3 (n = 71)	*p*
Total calories	2098 ± 650	2125 ± 780	2190 ± 840	0.610
Proteins (%E/d)	19.1 ± 4.9	18.6 ± 4.9	20.6 ± 3.8	0.421
Fat (%E/d)	36.4 ± 6.2	37.4 ± 5.6	35.8 ± 9.1	0.720
Carbohydrates (%E/d)	49.4 ± 6.3	51.6 ± 5.3	52.9 ± 7.6	0.456
Fiber (g/d)	23.3 ± 3.1	21.6 ± 4.2	22.8 ± 6.9	0.520

Values are presented as means ± standard deviations.

**Table 3:** Biochemical profile of participants stratified by betaine consumption.

Variable	Group 1 (n = 70)	Group 2 (n = 71)	Group 3 (n = 71)	*p*
Fasting glucose (mg/dL)	92.4 ± 9.4	95.1 ± 10.6	94.6 ± 11.4	0.260
Total cholesterol (mg/dL)	202 ± 37	199 ± 38	180 ± 31	**0.002** ^a^
HDL-c (mg/dL)	46.5 ± 12.9	44.5 ± 16.4	42.6 ± 13.8	0.322
Non-HDL-c (mg/dL)	154 ± 40	151 ± 37	137 ± 30	**0.017** ^b^
LDL-c (mg/dL)	134 ± 35	129 ± 34	118 ± 32	**0.028** ^c^
Triglycerides (mg/dL)	113 ± 76	108 ± 55	105 ± 52	0.734

Values are presented as means ± standard deviations. HDL-c: high-density lipoprotein cholesterol; no-HDL-c: cholesterol no HDL; LDL-c: low-density lipoprotein cholesterol. ^a^ Post hoc tests: group 1 vs. group 3, *p* = 0.001; group 2 vs. group 3, *p* = 0.008. ^b^ Post hoc tests: group 1 vs. group 3, *p* = 0.021. ^c^ Post hoc tests: group 1 vs. group 3, *p* = 0.026. Bold numbers indicate *p* < 0.05.

## Data Availability

The data supporting the findings of this research are available on request from the corresponding author.
